# Resorcinol-, catechol- and saligenin-based bronchodilating β2-agonists as inhibitors of human cholinesterase activity

**DOI:** 10.1080/14756366.2017.1326109

**Published:** 2017-06-02

**Authors:** Anita Bosak, Anamarija Knežević, Ivana Gazić Smilović, Goran Šinko, Zrinka Kovarik

**Affiliations:** a Institute for Medical Research and Occupational Health, Zagreb, Croatia;; b Ruđer Bošković Institute, Zagreb, Croatia

**Keywords:** Acetylcholinesterase, butyrylcholinesterase, adrenaline, salmeterol, terbutaline

## Abstract

We investigated the influence of bronchodilating β2-agonists on the activity of human acetylcholinesterase (AChE) and usual, atypical and fluoride-resistant butyrylcholinesterase (BChE). We determined the inhibition potency of racemate and enantiomers of fenoterol as a resorcinol derivative, isoetharine and epinephrine as catechol derivatives and salbutamol and salmeterol as saligenin derivatives. All of the tested compounds reversibly inhibited cholinesterases with *K*_i_ constants ranging from 9.4 μM to 6.4 mM and had the highest inhibition potency towards usual BChE, but generally none of the cholinesterases displayed any stereoselectivity. Kinetic and docking results revealed that the inhibition potency of the studied compounds could be related to the size of the hydroxyaminoethyl chain on the benzene ring. The additional π–π interaction of salmeterol’s benzene ring and Trp286 and hydrogen bond with His447 probably enhanced inhibition by salmeterol which was singled out as the most potent inhibitor of all the cholinesterases.

## Graphical Abstract

**Figure:**
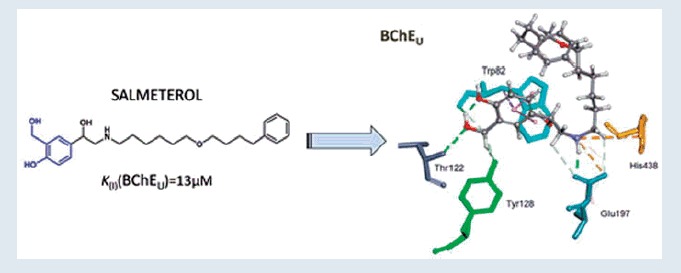

## Introduction

The transfer of nervous impulses via acetylcholine (ACh) depends on a series of well-tuned receptors, enzymes that take part in the synthesis and hydrolysis of ACh and a series of transporters^1^. In chronic inflammatory respiratory diseases (asthma and chronic obstructive pulmonary disease), ACh levels increase, which leads to the contraction of the smooth bronchial muscle tissue, intensified mucous secretion, chronic coughing and immune stimulation^2^^,^^3^. The degradation of ACh in the organism takes place through the activity of acetylcholinesterase (AChE), which thus fulfils its physiological role. This role is shared by the closely related enzyme butyrylcholinesterase (BChE), an enzyme that does not possess a known physiological substrate, but does take part in the bioconversion of several xenobiotics, in the metabolism of lipids and lipoproteins and serves as a backup enzyme in acetylcholine hydrolysis when AChE is inhibited^4–7^. Both AChE and BChE are crucial in the treatment of neurodegenerative disorders such as *Myasthenia gravis*, Alzheimer and Parkinson’s disease, since generally all drugs in use act by inhibiting AChE and BChE activity^6^^,^^8^. The activity of both enzymes depends on factors such as ethnicity, exposure to anticholinergic reagents such as organophosphorus and carbamate pesticides, nerve agents and anticholinergic drugs^4^^,^^9^^,^^10^. BChE activity is additionally affected by flavonoids or natural anticholinesterase toxins in food and drugs whose bioconversion involves the action of BChE, like echothiophate, eye drops for glaucoma treatment, anti-Alzheimer drugs or the bronchodilator bambuterol^4^^,^^11–15^. However, the efficacy of any drug that targets BChE activity can be affected by the human *BCHE* gene polymorphism. At present, more than 56 mutations of the human *BCHE* gene have been identified^16^, and different catalytic properties or lower enzyme levels than of usual BChE have been confirmed for several BChE variants; for example, individuals homozygous for atypical butyrylcholinesterase can experience prolonged apnoea if the muscle relaxant succinylcholine or mivacurium are administered^16^^,^^17^.

β-Adrenergic agonists are potent bronchodilators widely used in the management of bronchial asthma. These agents interact with β2-adrenergic receptors on the smooth muscle of bronchial tissue relieving bronchospasms and reducing airway resistance. Beside the bronchial tissue β-adrenergic agonists also act on other tissues where signals to other cells are regulated by acetylcholine, like smooth muscles of the vascular system, intestines and uterus. Recent studies have shown that oral intake of salbutamol and ephedrine appeared to be effective supplement treatments in severe cases of acetylcholine receptor (AChR)^18^, while chronic treatment with certain β2-agonists relieved neuropathic pain^19^. Also, recent studies demonstrated that some bronchodilators could be used as additional antidotes in the treatment of poisoning by organophosphates, either in accidental poisonings by organophosphate pesticides or following acts of terrorism using nerve agents^20–23^. Organophosphates act through the inhibition of AChE consequently leading to a collapse of the respiratory system. The studies of the effects of AChE and BChE inhibition in KO mice have shown that the respiratory function is more sensitive to anticholinesterases in AChE heterozygotes than in wild-type mice, suggesting that BChE is an important enzyme for the respiratory function and should be left unaffected by medications that have the ability to reduce cholinesterase activity^24^. Ventilatory, inflammatory and lethal effects of sarin may be limited by the use of epinephrine and oxygen^20^, while the protective activity of salbutamol was demonstrated in diisopropylfluorophosphate (DFP) poisoning^21^^,^^22^. Also, treatment with Combivent (a drug consisting of ipratropium and salbutamol) improved the respiratory outcome after exposure to soman but did not influence the final lethal outcome^23^.

Among the various chiral drugs, one enantiomer is often more active than another in treating a medical condition. An example is the (*R*)-enantiomer of salbutamol, which is approximately 80 times more potent as a β-adrenoceptor agonist than (*S*)-enantiomer and the administration of pure (*R*)-salbutamol improved therapeutic activity and reduced side effects^25^. Similar *in vivo* results were obtained with enantiomers of terbutaline^26^. AChE and BChE are stereoselective enzymes in the interaction with various esters^7^^,^^9^^,^^27^^,^^28^. In interaction with bambuterol, which in its racemic form is in use as a prodrug of terbutaline, both enzymes prefer (*R*)- over (*S*)-bambuterol^7^^,^^27^^,^^28^.

**Figure 1.**
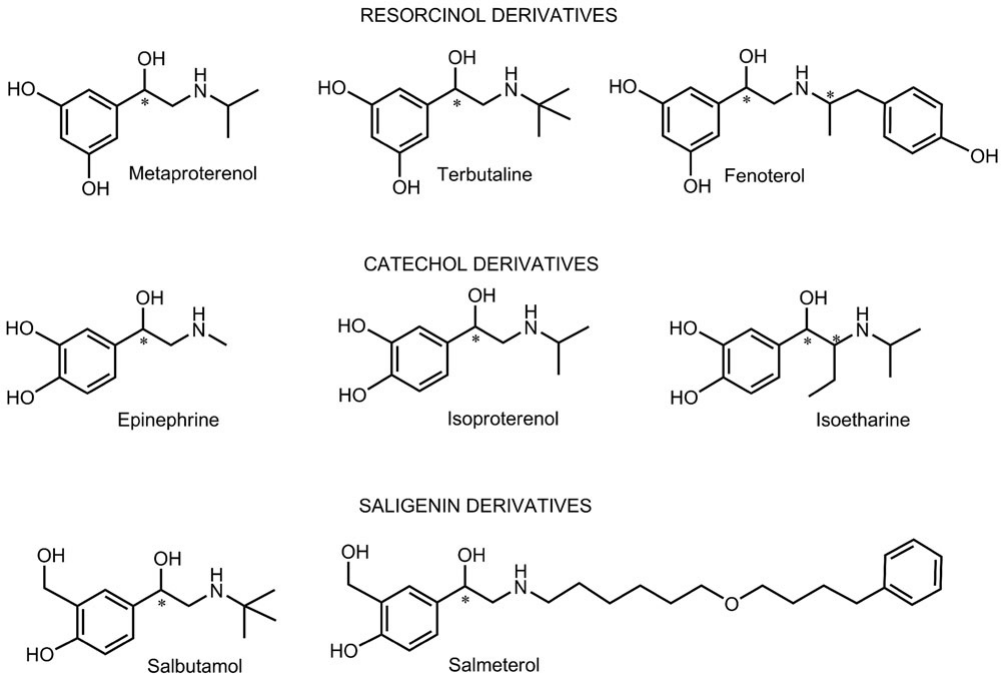
The chemical structure of the studied resorcinol, catechol and saligenin derivatives. Asterisk denotes a chiral carbon atom.

In our previous studies, we demonstrated that some derivatives of resorcinol were reversible inhibitors of human cholinesterases^9^^,^^28^^,^^29^. In this study, we extended our research on other β-agonists with a resorcinol-, catechol- and saligenin-containing structure. Here, we determined the inhibition potency of fenoterol as a resorcinol derivative, isoetharine and epinephrine as catechol derivatives, and salbutamol and salmeterol as saligenin derivatives (Figure 1) towards human AChE and three variants of human BChE (usual, atypical and fluoride resistant). The systematic structural differences between compounds and their inhibition potency towards certain cholinesterases were analysed by molecular modelling.

## Material and methods

### Chemicals

Racemic epinephrine, isoetharine, fenoterol and terbutaline, as well as (*R*)-epinephrine, were purchased (Sigma-Aldrich, St. Louis, MO) in the form of salts and converted to hydrochloride salts (detailed in Supplementary material). All of the chemicals, reagents and solvents for the preparation of resorcinol, catechol and saligenin derivatives were purchased from commercial sources and used without further purification. Acetylthiocholine iodide (ATCh), propionylthiocholine iodide (PTCh) and 5,5′-dithiobis(2-nitrobenzoic acid) (DTNB) were purchased from Sigma-Aldrich. All of the inhibitors and substrates were dissolved in water, while DTNB was dissolved in 0.1 M sodium phosphate buffer (pH 7.4). Only salmeterol was dissolved in ethanol due to its low solubility, while all further dilutions were made in water.

### Enzymes

Human native serum was used as a source of BChE. Recombinant human AChE was a gift from Dr Florian Nachon (Département de Toxicologie, IRBA, France). BChE variants were confirmed earlier as described previously^30^^,^^31^. The usual BChE was collected from a female donor at the Institute for Medical Research and Occupational Health, Zagreb, Croatia (IMROH); the atypical BChE was a gift from Dr Oksana Lockridge (University of Nebraska Medical Centre, Eppley Institute, Omaha, NE), and specimens of the fluoride resistant BChE were a gift by Dr Robert T. Evans (Cholinesterase Investigation Unit, St’ James University Hospital, Leeds, Great Britain). Enzyme dilutions were done in phosphate buffer and those of recombinant AChE with the addition of 0.01% BSA. This study was reviewed and approved by the Ethics Committee of IMROH.

### Synthesis

Racemic salbutamol and salmeterol were synthesised starting from methyl 5-acetylsalicylate (Sigma-Aldrich) according to literature procedures described previously^32^^,^^33^.

The enantiomers of salbutamol and (*R*)-terbutaline were prepared from their racemic form using diastereomeric salt crystallization method^32^^,^^34^. The resolving agent for (*R*)-terbutaline and (*R*)-salbutamol precursor was (+)-O,O′-di-*p*-toluoyl-d-tartaric acid and (−)-O,O′-di-*p*-toluoyl-l-tartaric acid for a precursor for (*S*)-salbutamol. Enantiomeric excess (ee) for the obtained enantiomers was determined using a brush-type chiral stationary phase previously synthesised in our laboratory (CSP 1)^35^. The obtained ee were 99%, 96% and 92% for (*S*)-salbutamol, (*R*)-terbutaline and (*R*)-salbutamol, respectively.

Enantiomers of fenoterol and salmeterol were resolved using semipreparative chiral chromatography. The columns used were CHIRALLICA PST-4 (AD-type column; Chirallica d.o.o., Croatia) and CHIRALLICA PST-2 (OJ-type column; Chirallica d.o.o., Croatia) for fenoterol and salmeterol, respectively. The obtained ee were 98%, 96%, 97% and 86% for (*S,S*)-fenoterol, (*R,R*)-fenoterol, (*R*)-salmeterol and (*S*)-salmeterol, respectively. The absolute configuration of enantiomers was determined by comparison of the elution order of these compounds on AD and OJ columns with literature data^36^^,^^37^ (detailed in Supplementary).

### Reversible inhibition

Enzyme activities were measured in 0.1 M phosphate buffer, pH 7.4 at 25 °C, on CARY 300 spectrophotometer equipped with temperature controller (Varian Inc., Australia) by Ellman method at 412 nm^38^. ATCh was used as a substrate for AChE and PTCh as a substrate for BChE. The reaction mixture contained DTNB (final concentration 0.3 mM), buffer, enzyme suspension, inhibitor (0.5–10 mM) and ATCh or PTCh (0.025–1.0 mM). Inhibition of enzymes was determined by measuring the decrease in enzyme activity in the presence of inhibitors. The baseline activity was determined in the presence of water since the proportion of ethanol in AChE and BChE activity measurement was up to 0.08% and 0.16%, respectively, which does not affect the enzyme activity.

The dissociation constant for an enzyme–inhibitor complex *K*_(I)_ was evaluated from the impact of substrate concentrations [*S*] on the degree of inhibition applying the Hunter–Downs equation^17^^,^^39^:

$$K_{i,\mathrm{app}}=\frac{v_{i}}{v_{0}-v_{i}}\cdot \left[I\right]=K_{\left(I\right)}+\frac{K_{\left(I\right)}}{K_{\left(S\right)}}\cdot \left[S\right]$$

where *K*_i,app_ is an apparent enzyme-reversible inhibitor complex dissociation constant obtained at a given substrate concentration [*S*] in the presence of inhibitor concentration [*I*] and calculated from the enzyme activities *v*_0_ and *v*_i_ measured without inhibitor and in the presence of inhibitor concentration [*I*], respectively^15^. *K*_(I)_ is the enzyme-reversible inhibitor complex dissociation constant and can stand for a complex formed at the catalytic site or for a complex formed at the peripheral site of the enzyme. *K*_(S)_ is the enzyme-substrate dissociation constant that could be approximated to the Michaelis constant or to the enzyme-substrate dissociation constant when two molecules of the substrate are bound to the enzyme.

### Molecular modelling

The docking of the studied compounds was performed using the Accelrys DiscoveryStudio 2.1 CDOCKER docking protocol and CHARMm force field. For the structural model of AChE, we used a crystal structure deposited in the Protein Data Bank, PDB ID 1B41^40^ and PDB ID 2PM8 as a model for usual BChE^41^. Models for atypical and fluoride-resistant BChE were constructed using the 2PM8 structure as a model in which Asp70 was replaced with Gly as a model for atypical BChE, and Thr243 with Met or Gly390 with Val as models of fluoride-resistant BChE. All of the constructed models were minimised using the CHARMm force field according to the docking protocol described previously^9^^,^^42^. As a result of molecular docking, a set of 20 possible poses per one compound and enzyme pair was analysed and the pose with the highest CDOCKER interaction energy was selected for further analysis.

## Results and discussion

The tested bronchodilating β2-agonists were divided into three groups according to the disposition of their hydroxyl groups on the benzene ring, resembling the structural feature of resorcinol, catechol and saligenin (Figure 1). The hydroxyl groups at the benzene were in the *meta* position in the resorcinol group, and in the *ortho* position in the catechol group. In the saligenin group, the hydroxyl group and the hydroxymethyl group were in the *ortho* position. Derivatives of each group differed in the size of the hydroxyaminoethyl chain attached to the benzene ring.

### Inhibition of human AChE

**Figure 2.**
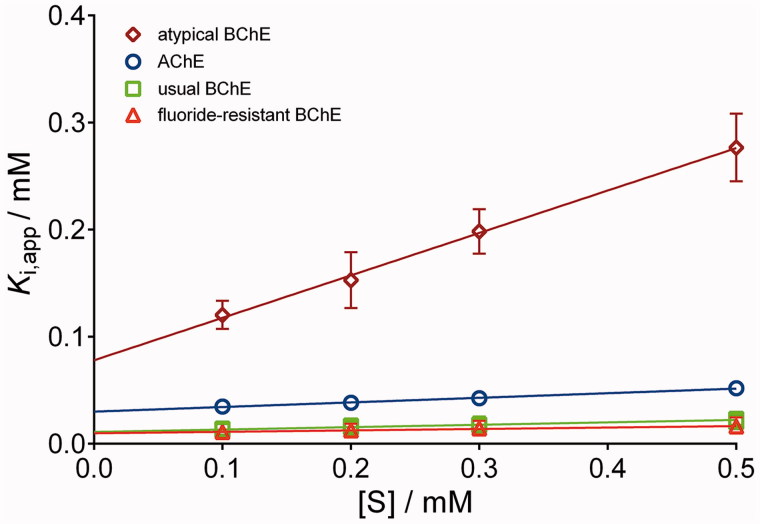
Reversible inhibition of human AChE and three BChE variants (usual, atypical and fluoride resistant) by racemic salmeterol. Points indicate the average apparent enzyme-inhibitor constant *K*_i,app_ (±SE) determined at a given substrate concentration (*S*) according to the Hunter–Downs equation. The lines are the result of linear regression analysis where the *y*-intercept represents the enzyme-inhibitor dissociation constant, *K*_(I)_.

Kinetic studies revealed that all of the studied bronchodilating β2-agonists reversibly inhibited AChE (Figure 2, an example of inhibition by racemic salmeterol), with enzyme-inhibitor dissociation constants (*K*_(I)_) ranging between 30 μM and 6.4 mM (Table 1), classifying these compounds as low-potency inhibitors^29^^,^^43^.

**Table 1.** Reversible inhibition of human AChE and three BChE variants (usual, atypical and fluoride resistant) by bronchodilating β2-agonists.

	*K*_(I)_/mM			
	AChE	usual BChE	atypical BChE	fluoride-resistant BChE
Resorcinol derivatives				
Metaproterenol^b^	3.1 ± 1.2	0.55 ± 0.11	6.1 ± 0.7	0.87 ± 0.18
Terbutaline	4.8 ± 0.2	0.18 ± 0.15^a^	3.3 ± 0.7^a^	0.30 ± 0.32^a^
Fenoterol	0.86 ± 0.05	0.024 ± 0.001	0.63 ± 0.03	0.061 ± 0.017
Catechol derivatives				
Epinephrine	6.4 ± 1.1	3.7 ± 1.2	1.6 ± 1.0	3.8 ± 1.5
Isoproterenol^b^	2.5 ± 1.0	0.47 ± 0.21	1.6 ± 0.3	0.99 ± 0.12
Isoetharine	0.21 ± 0.04	0.031 ± 0.003	0.26 ± 0.05	0.086 ± 0.004
Saligenin derivatives				
Salbutamol	2.0 ± 0.2	0.071 ± 0.006	1.4 ± 0.2	0.19 ± 0.02
Salmeterol	0.030 ± 0.002	0.013 ± 0.001	0.086 ± 0.018	0.0098 ± 0.0023

The enzyme-inhibitor complex dissociation constant (*K*_(I)_± SE) was determined by linear regression from *K*_i,app_ constants obtained from at least two experiments.

a Reference^29^.

b Reference^9^.

Ranges of *K*_(I)_ constants of resorcinol, catechol and saligenin derivatives were very similar, indicating that the inhibition potency of the tested bronchodilating β2 agonists towards human AChE does not depend on the disposition of hydroxyl groups on the benzene ring. However, it seems that the inhibition potency of the tested β2-agonists depends on the size of the hydroxyaminoethyl chain attached to the benzene ring. For example, in the resorcinol group, metaproterenol and terbutaline differ only in the end of hydroxyaminoethyl chain (isopropyl in metaproterenol versus tert-butyl in terbutaline) exhibiting very similar inhibition, while the enlargement of the hydroxyaminoethyl chain with the hydroxybenzyl group, as it is a fenoterol, caused a fivefold increase in inhibition potency. In the catechol group, an elongation of the hydroxyaminoethyl chain from methyl to isopropyl lead to a 2-fold increase in inhibition potency of isoproterenol compared to epinephrine. Additional substitution on the hydroxyaminoethyl chain with the ethyl group, as in isoetharine, caused a further 12-fold higher inhibition potency compared to isoproterenol. The largest, 67-fold, increase in inhibition potency was observed in the saligenin group between salbutamol and salmeterol, where the largest difference in the size of hydroxyaminoethyl chain was. Additionally, when compounds with identic hydroxyaminoethyl chain but different disposition of hydroxyl group on benzene ring (metaproterenol vs. isoproterenol) were compared, no difference in inhibition potency was observed. Similarly two times higher inhibition potency of salbutamol, compared to terbutaline, can be attributed to the substitution of one hydroxyl group on benzene ring with the hydroxymethyl group. From all of the tested bronchodilating β2-agonists, salmeterol was singled out as the most potent inhibitor with an inhibition potency similar to that of flavonoids quercetin and myricetin^44^.

**Figure 3.**
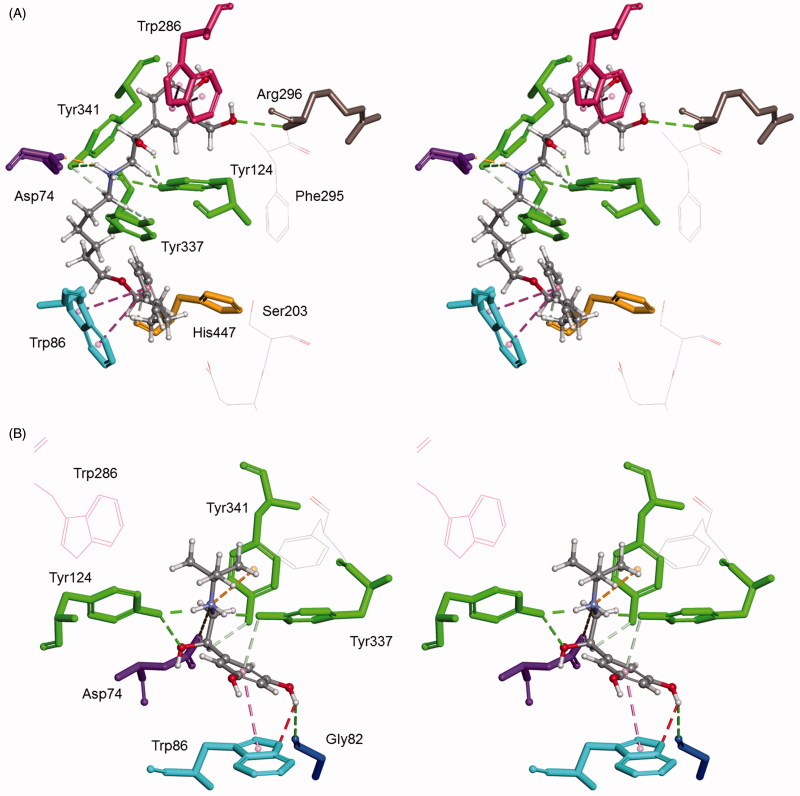
Computational molecular modelling of *β*2-agonists and human AChE (PDB ID 1B41). Stereo view of salmeterol (A) and terbutalin (B) in the active site of AChE. Ligands create H-bonds with residues (green-dashed lines). Hydrophobic *π–π* interactions with aromatic residues are represented as cyan-dashed lines and *π*–cation interactions are represented as orange-dashed lines. The hydrogen atoms of the residues have been omitted for better visibility.

The inhibition potency correlated well with the docking results expressed here as the interaction energy of the bronchodilating β2-agonists − AChE complex (Table 2). The highest interaction energy was that of salmeterol, the most potent AChE inhibitor. All of the predicted non-covalent interactions of bronchodilating β2-agonists with amino acid residues defining the AChE active site gorge are summarised in Table 3. The analysis of these interactions showed that, regardless of the size of the hydroxyaminoethyl chain on the benzene ring, the chain was always directed to the peripheral site of the active centre gorge of the enzyme. This orientation was due to the formation of hydrogen bonds with Tyr124 and Asp74 (Figure 3(A,B)). These interactions, together with interactions with tyrosines 337 and 341 and the position of the benzene ring relative to Trp86, were those that governed the positioning of the tested compounds into the active site of AChE. It seems that epinephrine was the weakest inhibitor of AChE merely because of the lack of stabilisation by hydrogen bonds with tyrosine residues 337 and 341. The difference between the inhibition potency of particular β2-agonists of the resorcinol, catechol and saligenin groups, was attributable to additional hydrogen bonds and hydrophobic interactions between the hydroxyaminoethyl chain and residues (Table 3). Salmeterol’s high inhibition potency could have been due to the additional π–π interaction of salmeterol’s benzene ring and Trp286 and hydrogen bond with His447 (Figure 3(A)).

**Table 2.** The highest CDOCKER interaction energy (CIE) between 20 poses per one bronchodilating *β*2-agonists − cholinesterase pair.

	—CIE (kJ/mol)		
	AChE	usual BChE	atypical BChE
Resorcinol derivatives			
Metaproterenol	178	149	178
Terbutaline	170	168	142
Fenoterol	245	182	231
Catechol derivatives			
Epinephrine	169	146	190
Isoproterenol	186	181	138
Isoetharine	203	212	204
Saligenin derivatives			
Salbutamol	178	169	156
Salmeterol	323	285	267

**Table 3.** List of bronchodilating *β*2-agonists − cholinesterase interactions.

	AChE	Usual BChE	Atypical BChE
Resorcinol derivatives			
Metaproterenol	H-bond: Asp74, Tyr124, Ser125, Glu202, Ser203	H-bond: Asp70, Trp82, Glu197	H-bond: Gly115, Tyr128, Glu197, Ser198, His438
	π–π: Trp86	π–π: Trp82	π–cation: Trp82
Terbutaline	H-bond: Asp74, Gly82, Tyr124, Tyr337, Tyr341	H-bond: Glu197, His438	H-bond: Glu197, Leu286
	π–π: Trp86	π–cation: Trp82	
	π–cation: Tyr341	π–alkyl: Ala328	
Fenoterol	H-bond: Asp74, Tyr124, Ser125, Tyr337, Tyr341,	H-bond: Asp70, Tyr128, Glu197, His438	H-bond: Thr120, Tyr128, Glu197, Tyr332, His438
	π–π: Trp86, Trp286, Tyr341	π–cation: Trp82 π–alkyl: Ala328	π–cation: Trp82, His438π–alkyl: Ala328
Catechol derivatives			
Epinephrine	H-bond: Asp74, Gly122, Tyr124, Ser125, Glu202, Ser203, Tyr337, Tyr341	H-bond: Glu197, His438, Gly439	H-bond: Trp82, Gly121, Tyr128, Glu197, Ser198, His438
	π–π: Trp86	π–π: Trp82	π–π: His438 π–σ: Gly116
Isoproterenol	H-bond: Asp74, Tyr124, Phe295, Tyr337, Tyr341,	H-bond: Asp70, Ser79, Glu197, Tyr332, His438	H-bond: Tyr440
	π–π: Trp286, Tyr341	π–cation: Trp82, His438	π–π: Trp82 π–alkyl: Ala328
Isoetharine	H-bond: Tyr124, Tyr133, Glu202, Tyr337	H-bond: Glu197, Ser198, Leu286, His438	H-bond: Thr120, Glu197, Ser198, His438
	π–π: Trp86	π–π: His438	π–π: His438, Gly116, Gly117
	π–alkyl: Tyr124, Phe297	π–alkyl: Trp82	π–alkyl: Trp82
Saligenin derivatives			
Salbutamol	H-bond: Asp74, Gly121, Tyr124, Tyr337	H-bond: Asp70, Asn83, His438	H-bond: Trp82, Gly121
	π–π: Trp86, Trp337	π–cation: Trp82	π–π: Trp82
	π–cation: Tyr341		π–σ: Gly116
Salmeterol	H-bond: Asp74, Tyr124, Phe295, Arg296, Tyr337, Tyr341, His447	H-bond: Trp82, Thr122, Tyr128, Glu197, His438	H-bond: Trp82, Thr120, Gly121, Glu197
	π–π: Trp86, Trp286	π–π: Trp82	π–π: Trp82, His438
		π–cation: His438	

### Inhibition of human BChE variants

**Figure 4.**
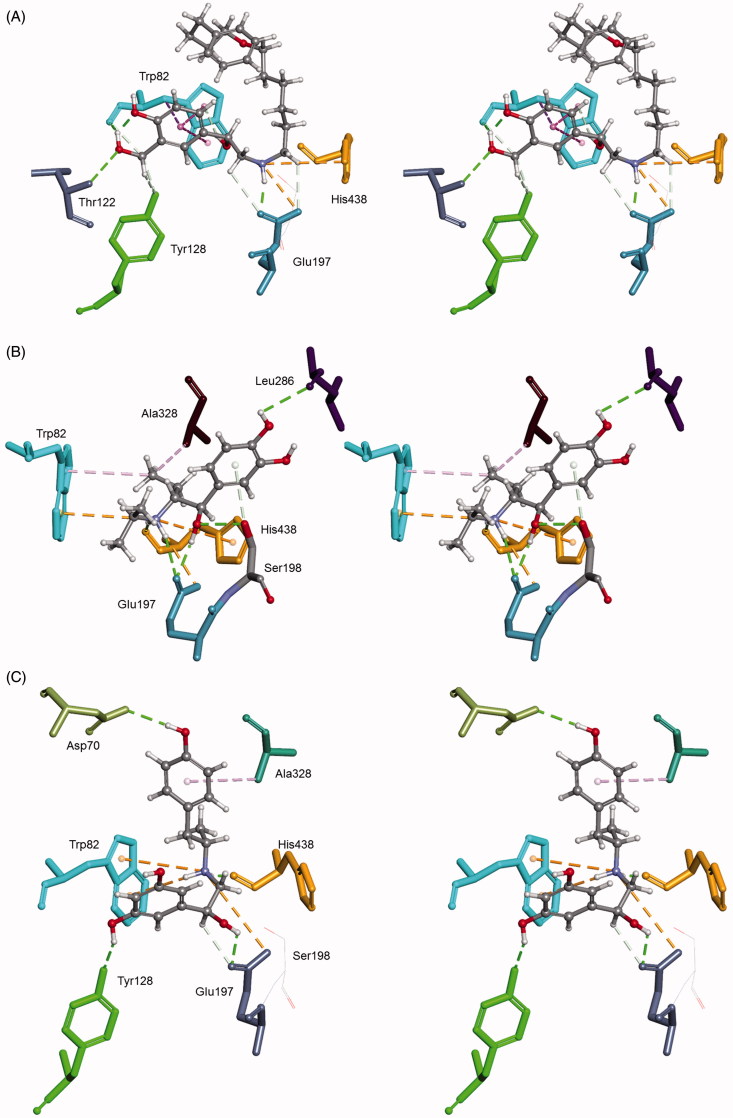
Computational molecular modelling of *β*2-agonists and human BChE (PDB ID 2PM8). Stereo view of salmeterol (A), isoetharine (B) and fenoterol (C) in the active site of usual BChE. Ligands create H bonds with residues (green dashed lines). Hydrophobic π–π interactions with aromatic residues are represented as cyan-dashed lines and π–cation interactions are represented as orange-dashed lines. The hydrogen atoms of the residues have been omitted for better visibility.

The studied bronchodilating β2-agonists reversibly inhibited all BChE variants (Figure 2, an example of inhibition by racemic salmeterol), with *K*_(I)_ constants ranging between 9.8 μM and 3.8 mM (Table 1). Generally, atypical and fluoride-resistant variants were up to 26 and 2.8 times, respectively, more weakly inhibited than usual BChE. The fluoride-resistant BChE has been characterised by an amino acid substitution changing T243M and G390V that are far from the enzyme-active centre but could cause conformational changes resulting in a lower inhibition potency of positively charged ligands^45^. The decrease of inhibition potency towards atypical BChE compared to usual BChE could be a consequence of the natural mutation of aspartate 70 to glycine (D70G), a residue located in the peripheral site of the active centre of BChE and close to the entrance of the active centre gorge. The inhibition potency of fenoterol, isoetharine and salmeterol towards usual BChE was up to 23, 120 and 5.5 times higher, respectively, than the potency of other resorcinol-, catechol- and saligenin-based compounds. The same trend of inhibition potency was observed for atypical and fluoride-resistant variants (Table 1). This indicates a positive correlation between the size of the hydroxyaminoethyl chain and the inhibition potency toward the BChE variants, similarly to our observation for AChE inhibition. Out of the eight bronchodilating β2-agonists tested, salmeterol was the most potent inhibitor of all BChE variants with a constant similar to that for donepezil, a drug used to treat Alzheimer’s disease^45^. The kinetic results correlated well with the docking results (Table 2); the higher the inhibitory potency of the compound, the higher the CDOCKER interaction energy. It seems that the inhibition potency of compounds with resorcinol-, catechol- or saligenin-based compounds towards usual BChE was primarily a consequence of cation–π interaction involving Trp82 (Table 3) that enabled their position and orientation in the active site of BChE allowing additional interactions such as hydrogen bonds with Glu197 and His438 residues (Figure 4(A–C)). The highest inhibition potency of salmeterol could be attributed to the additional *π–π* interaction with Trp82, which anchored the saligenin part deeper in the active site and directed the hydrocarbon chain towards the exit of the active site of the usual BChE. This position seemed optimal for the inhibition of BChE.

### Inhibition selectivity

Due to a difference in residues lining the active site of AChE and BChE, these two enzymes have different reactivity toward substrates, inhibitors and ligands^9^^,^^10^^,^^44^^,^^46^. Therefore, it is important to evaluate the selectivity of compounds that either targets the activity of AChE, as it is with some drugs for treating neurodegenerative disorders like Alzheimer’s disease^46^ or prodrugs whose bioconversion includes the action of BChE, as in the case of bambuterol^9^^,^^11^. The selectivity of the studied bronchodilating β2-agonists was determined from the ratio of the corresponding *K*_(I)_ constants for AChE and BChE. Generally, all of the compounds were more potent inhibitors of BChE than of AChE. The most selective inhibitor was fenoterol, followed by salbutamol and terbutaline that inhibit usual BChE 36, 28 and 27 times more potently than AChE, respectively. It is worthy to mention that these β2-agonists are currently the most frequently used bronchodilators. Catechol derivatives were the least selective inhibitors, out of which epinephrine showed the lowest selectivity. Salmeterol, the most potent inhibitor of all of the tested cholinesterases, displayed only a two times higher potency towards BChE than toward AChE. In view of the BChE polymorphism, inhibition of fluoride-resistant BChE is similarly selective as that of usual BChE, while no selectivity in the inhibition of the atypical variant was determined.

### Stereoselectivity of human AChE and usual, atypical and fluoride-resistant BChE

The tested compounds are chiral molecules (Figure 1) and the stereoselectivity of cholinesterases could have been expected as a result of a difference in the binding of enantiomers. The stereoselectivity was determined from the ratio of *K*_(I)_ constants determined for the (*R*) and (*S*) enantiomers of fenoterol, salbutamol and salmeterol (Table 4). For terbutaline, epinephrine and isoproterenol, stereoselectivity was estimated by comparing the *K*_(I)_ constants obtained for racemate with those for the (*R*) enantiomer (Table 4). Irrespectively of whether stereoselectivity was determined or just estimated, for most of the compounds, neither AChE nor BChEs displayed any stereoselectivity; the exception was AChE and atypical BChE preferring 2- and 3-fold (*R*)-terbutaline over its racemate, respectively. The lack of cholinesterase stereoselectivity was probably the consequence of interactions with residues that did not involve the chiral centre of the tested compounds. Indeed, the molecular modelling of complexes demonstrated that the stabilisation of the compounds into the active site of the enzymes was based on other interactions (Figures 3 and 4).

**Table 4.** Reversible inhibition of human AChE and three BChE variants (usual, atypical and fluoride-resistant) by enantiomers of bronchodilating β2-agonists.

	*K*_(I)_/mM				
	AChE	Usual BChE	Atypical BChE		Fluoride-resistant BChE
Resorcinol derivatives					
(*R*)-terbutaline	2.0 ± 0.3	0.11 ± 0.03		1.0 ± 0.1	0.22 ± 0.05
(*R,R*)-fenoterol	0.54 ± 0.03	0.0094 ± 0.0008		0.13 ± 0.02	0.019 ± 0.004
(*S,S*)-fenoterol	0.35 ± 0.03	0.013 ± 0.001		0.10 ± 0.00	0.037 ± 0.005
Catechol derivatives					
(*R*)-epinephrine	3.2 ± 0.4	4.7 ± 1.8	12 ± 2		—
(*R*)-isoproterenol	2.0 ± 0.5	0.81 ± 0.13	1.4 ± 0.1		1.4 ± 0.2
Saligenin derivatives					
(*R*)-salbutamol	4.0 ± 0.3	0.19 ± 0.04	2.3 ± 0.2		0.30 ± 0.06
(*S*)-salbutamol	1.8 ± 0.1	0.31 ± 0.05	1.5 ± 0.2		0.30 ± 0.04
(*R*)-salmeterol	0.033 ± 0.003	0.015 ± 0.001	0.071 ± 0.019		0.031 ± 0.004
(*S*)-salmeterol	0.028 ± 0.006	0.015 ± 0.001	0.067 ± 0.013		0.020 ± 0.003

— not measured.

Enzyme-inhibitor complex dissociation constants (*K*_(I)_±SE) were determined by linear regression from *K*_i,app_ constants obtained from at least two experiments.

## Conclusions

Due to the important and versatile functions of cholinesterases in organisms, it is important to define the inhibition potency of compounds that reduce the level of their hydrolytic activity, especially for those in use as drugs. All of the tested β2-agonists are in use as bronchodilators, except isoetharine whose use was discontinued. Our results have shown that the compounds are reversible cholinesterase inhibitors that reduce human cholinesterase activity and therefore can affect their roles in an organism. Cholinesterases had the lowest affinity towards epinephrine, the hormone of adrenal glands and neurons that plays an important role in the organism during acute stress response. Interestingly, AChE also displayed low affinity towards terbutaline and salbutamol, compounds that are active components of the most commonly used bronchodilators. It should also be mentioned that the tested β2-agonists showed the highest inhibition potency towards usual BChE among the three variants of BChE. Moreover, our results show that the racemates of these compounds in terms of cholinesterase inhibition are safe for use since there was no stereoselectivity exhibited in the interaction with cholinesterases.

## Supplementary Material

IENZ_1326109_Supplementary_Material.pdf
