# Supplementary material for: Resorcinol-, catechol- and saligenin-based bronchodilating β2-agonists as inhibitors of human cholinesterase activity
Source: J Enzyme Inhib Med Chem. 2017 Jun 2;32(1):789–97. doi: 10.1080/14756366.2017.1326109 (PMC6445159; doi:10.1080/14756366.2017.1326109)
Supplement: IENZ_1326109_Supplementary_Material.pdf [file IENZ_A_1326109_SM9905.pdf]

Supporting Information

Resorcinol-, catechol- and saligenin-based bronchodilating β2-agonists as potential modulators of human cholinesterase activity

Anita Bosak, Anamarija Knežević, Ivana Gazić Smilović, Goran Šinko, Zrinka Kovarik

Content:

General considerations

Preparation of hydrochloride salts of compounds

Preparation of enantiomers

Chromatographic separation of fenoterol and salmeterol

HPLC chromatograms of racemic terbutaline, salbutamol, fenoterol, salmeterol, and determination of ee of (*R*)-terbutaline; (*R*)- and (*S*)-salbutamol; (*R,R*)- and (*S,S*)-fenoterol; (*R*)- and (*S*)-salmeterol

General Considerations

Racemic epinephrine hydrochloride, isoetharine mesylate, fenoterol hydrobromide, terbutaline hemisulfate, salbutamol and (−)-epinephrine were purchased from Sigma-Aldrich (USA).

Chiral HPLC analysis were performed using a Shimadzu Prominence System (Pump LC-20AT, DGU-20A5 Degasser, UV detector SPD-20A) or Knauer system (Pump Knauer 64, 4-Port Knauer Degasser, UV detector Knauer Variable Wavelength Monitor, Interface Knauer, and CD detector Jasco CD-2095.

Chiral separations were performed on a CHIRALLICA PST-4 (amylose tris(3,5-dimethylphenylcarbamate) coated on silica-gel, AD-type column) or a CHIRALLICA PST-2 (cellulose tris(4-methylbenzoate) coated on silica-gel, OJ-type column) from Chirallica d.o.o. (Croatia), dimensions 250 mm x 4.6 mm i.d. for analytical column and 250 mm x 8 mm i.d. for the semipreparative column. Enantiomeric excess of salbutamol enantiomers and (*R*)-terbutaline was determined using brush-type chiral stationary phase which was previously synthesized in our laboratory by covalently binding (*2S*)-N-(3,5-dimethylphenyl)-2-[(4-chloro-3,5-dinitrophenyl)carbonylamino]propan-amide to aminopropyl silica (**CSP 1**) (Konterc et al. 2003).

Preparation of hydrochloride salts of compounds

A few drops of 2 M NaOH solution were added to a solution of the racemic salts of isoetharine, fenoterol and terbutaline (100 mg) in water (2 mL). The solvent was removed under reduced pressure, 2-PrOH (2 mL) added and once more evaporated. To obtain a solid, 2-PrOH was added

(3 mL) and the resulting precipitate was filtered off. To the resulting filtrate, a few drops of the solution of HCl in 2-PrOH were added. The solvent was removed under reduced pressure which yielded a corresponding hydrochloride salt.

Preparation of enantiomers

(*R*)-terbutaline was prepared according to a known literature procedure from racemic terbutaline hemisulfate using (+)-*O*,*O*′-di-*p*-toluoyl-D-tartaric acid as an effective resolving agent (Liao et al. 2003). Enantiomeric excess (*ee* 96 %) was determined on **CSP 1** in the mobile phase hexane/THF/EtOH/DIPA/TFA = 75/20/5/0.1/0.1 (Figure 1), the flow rate was 1.0 ml/min, 280 nm, t_{R1} = 19.2 min, t_{R2} = 22.8 min. The obtained (*R*)-terbutaline was converted to hydrochloride salt using the above-mentioned procedure.

Enantiomers of salbutamol were prepared by reducing single enantiomers of 5-[2-[(1,1-dimethylethyl)amino]-1-hydroxyethyl]-2-hydroxybenzoate, precursors to salbutamol enantiomers (Gao and Zepp 1995). The resolution of the salbutamol precursor was accomplished with a chiral acid: (+)-*O*,*O*′-di-*p*-toluoyl-D-tartaric acid for the (*R*)-salbutamol precursor and (−)-*O*,*O*′-di-*p*-toluoyl-L-tartaric acid for the (*S*)-salbutamol precursor. Enantiomeric excess of salbutamol enantiomers was determined on **CSP 1** in the mobile phase hexane/2-PrOH/DIPA/TFA = 80/20/0.1/0.1, the flow rate was 2.0 ml/min, 220 nm, t_{R1} = 13.5 min, t_{R2} = 15.7 min and numbers *ee* 99 % for (*S*)-salbutamol and *ee* 92 % for (*R*)-salbutamol (Figure 2).

Chromatographic separation of fenoterol and salmeterol

Resolution of fenoterol enantiomers

Enantiomers of fenoterol were successfully resolved on an analytical CHIRALLICA PST-4 column (dimensions: 250 mm x 4.6 mm i. d.) using hexane/EtOH/DEA = 80/20/0.1 mobile phase (Figure 3), UV detection (λ = 220 nm), the flow rate was 1.0 ml/min, t_{R1} = 11.1 min, t_{R2} = 14.9 min. However, for the separation on the semi-preparative column (dimensions: 250 mm x 8 mm i. d.) with a flow rate of 5 mL/min, the best mobile phase proved to be hexane/EtOH/DEA = 85/15/0.1. (Figure 4). Racemic fenoterol (140 mg) was dissolved in the EtOH (2 mL) with a few drops of DEA. A total of 100 µl of this solution was injected, and after 20 runs, 140 mg of the racemic compound was resolved. After the removal of the solvent, the obtained enantiomers were converted to hydrochloride salts using 2-PrOH solution of hydrochloric acid. According to the literature, using the AD column, the first eluted enantiomer (*ee* 98 %) is (*S*, *S*)-fenoterol, while the second eluted enantiomer (*ee* 96 %) is (*R*, *R*)-fenoterol (Figure 5) (Beigi et al. 2006).

Resolution of salmeterol enantiomers

Enantiomers of salmeterol were resolved on an analytical CHIRALLICA PST-2 column (dimensions: 250 mm x 4.6 mm i. d.) using mobile phase hexane/EtOH/DEA = 90/10/0.1, the flow rate of 1 mL/min, UV detection (λ = 220 nm), t_{R1} = 23.3 min, t_{R2} = 30.8 min. The same mobile phase was used for separation on a semi-preparative column (dimensions: 250 mm x 8 mm i. d.) with a flow rate of 4 mL/min (Figure 6). Racemic salmeterol (100 mg) was dissolved in EtOH:hexane = 1:1 (2 mL). The 90 µl of this solution was injected, and after 22 runs, 100 mg of the racemic compound was resolved. According to the literature, using the OJ column, the first

eluted enantiomer (*ee* 97 %) is (*R*)-salmeterol, while the second eluted enantiomer (*ee* 86 %) is (*S*)-salmeterol (Coe et al. 2003).

Literature:

Kontrec, D.; Vinković, V.; Šunjić, V.; Schuiki, B.; Fabian, W. M. F.; Kappe, C. O. Enantioseparation of Racemic 4-Aryl-3,4-Dihydro-2(1H)-Pyrimidones on Chiral Stationary Phases Based on 3,5-Dimethylanilides of N-(4-Alkylamino-3,5-Dinitro)Benzoyl L-Alpha-Amino Acids. *Chirality* **2003**, *15*, 550-557. DOI: 10.1002/chir.10200

Liao, J.; Peng, X.; Zhang, J.; Yu, K.; Cui, X.; Zhu, J.; Deng, J. Facile Resolution of Racemic Terbutaline and a Study of Molecular Recognition Through Chiral Supramolecules Based on Enantiodifferentiating Self-Assembly. *Org. Biomol. Chem*. **2003**, *1*, 1080–1085. DOI: 10.1039/b211327a

Gao, Y.; Zepp, C. M. Enantioselective Preparation of Optically Pure Albuterol via Resolution of Methyl 5-[2-[(1,1-Dimethylethyl)Amino]-1-Hydroxyethyl]-2-Hydroxybenzoate with Ditoluoyltartaric Acid. US Patent No. 1995/005399765A. **1995**.

Beigi, F.; Bertucci, C.; Zhu, W.; Chakir, K.; Wainer, I. W.; Xiao, R.-P.; Abernethy, D. R. Enantioselective Separation and Online Affinity Chromatographic Characterization of R,R- and S,S-Fenoterol. *Chirality* **2001**, *8(6)*, 822-827. DOI: 10.1002/chir.20317

Coe, D. M.; Perciaccante, R.; Procopiou, P. A. Potassium Trimethylsilanolate Induced Cleavage of 1,3-Oxazolidin-2- and 5-Ones, and Application to the Synthesis of (*R*)-Salmeterol. *Org. Biomol. Chem*. **2003**, *1*, 1106–1111. DOI: 10.1039/b212454h

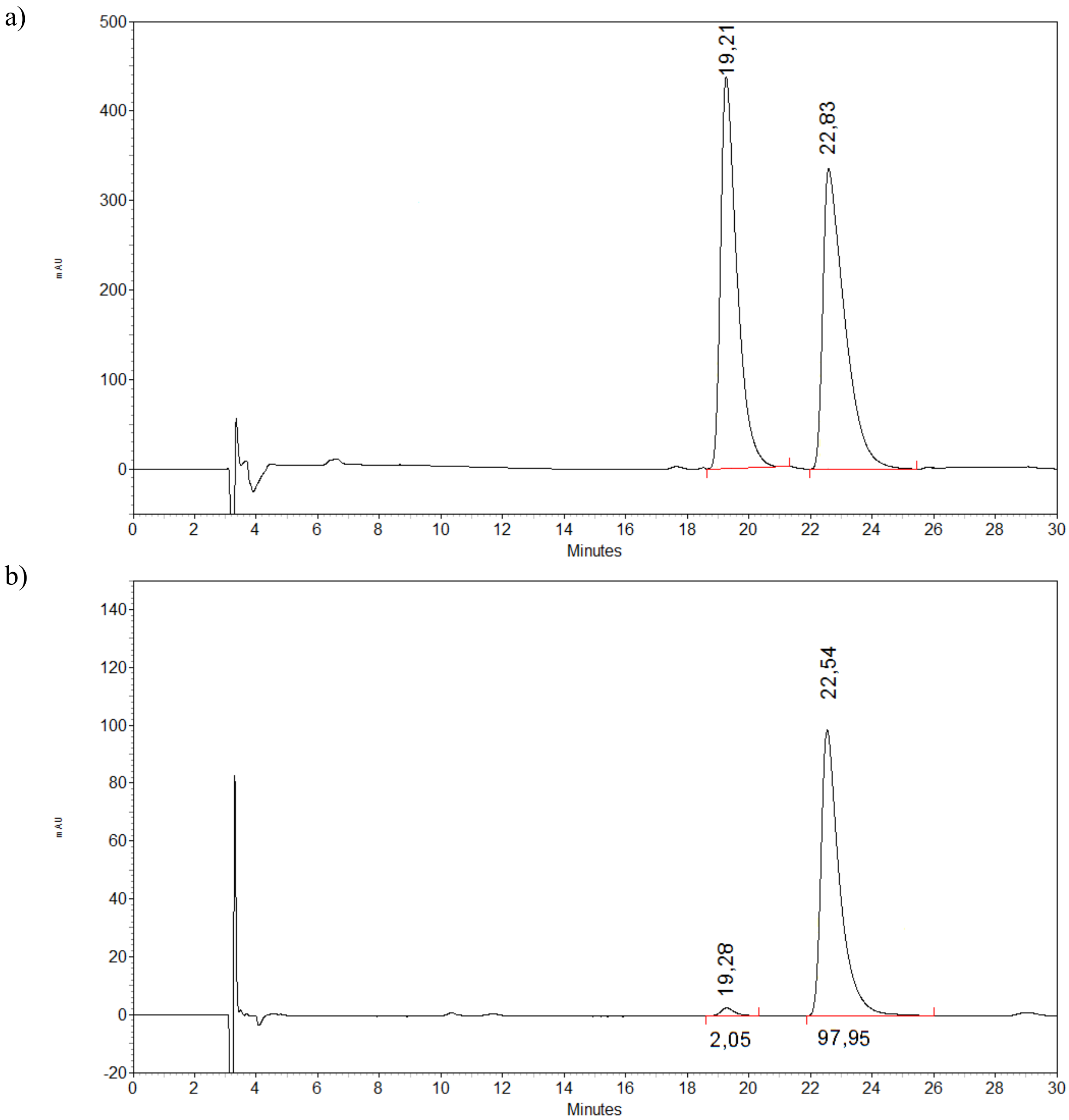

Figure 1. a) Chromatogram of racemic terbutaline on **CSP 1** and b) determination of *ee* for (*R*)-terbutaline on **CSP 1**. Mobile phase was hexane/THF/EtOH/DIPA/TFA = 75/20/5/0.1/0.1, the flow rate was 1.0 ml/min, 280 nm. The retention time of the peak is given above the corresponding peak and area percentage is given below.

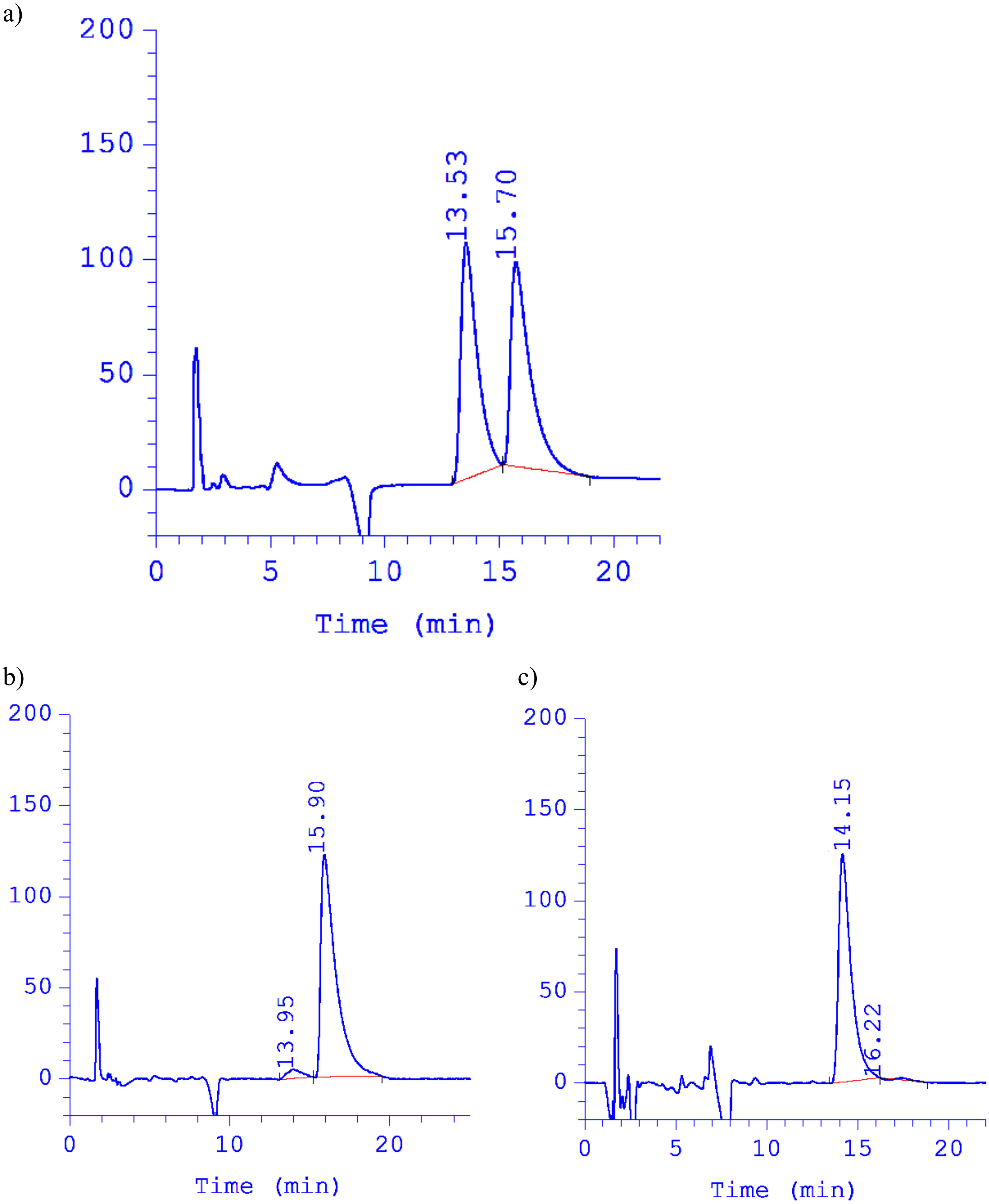

Figure 2. a) Chromatogram of racemic albuterol on **CSP 1** and b) determination of *ee* for (*R*)-salbutamol and c) (*S*)-salbutamol on **CSP 1**. Mobile phase was hexane/2-PrOH/DIPA/TFA = 80/20/0.1/0.1, the flow rate was 2.0 ml/min, 220 nm. The retention time of the peak is given above the corresponding peak.

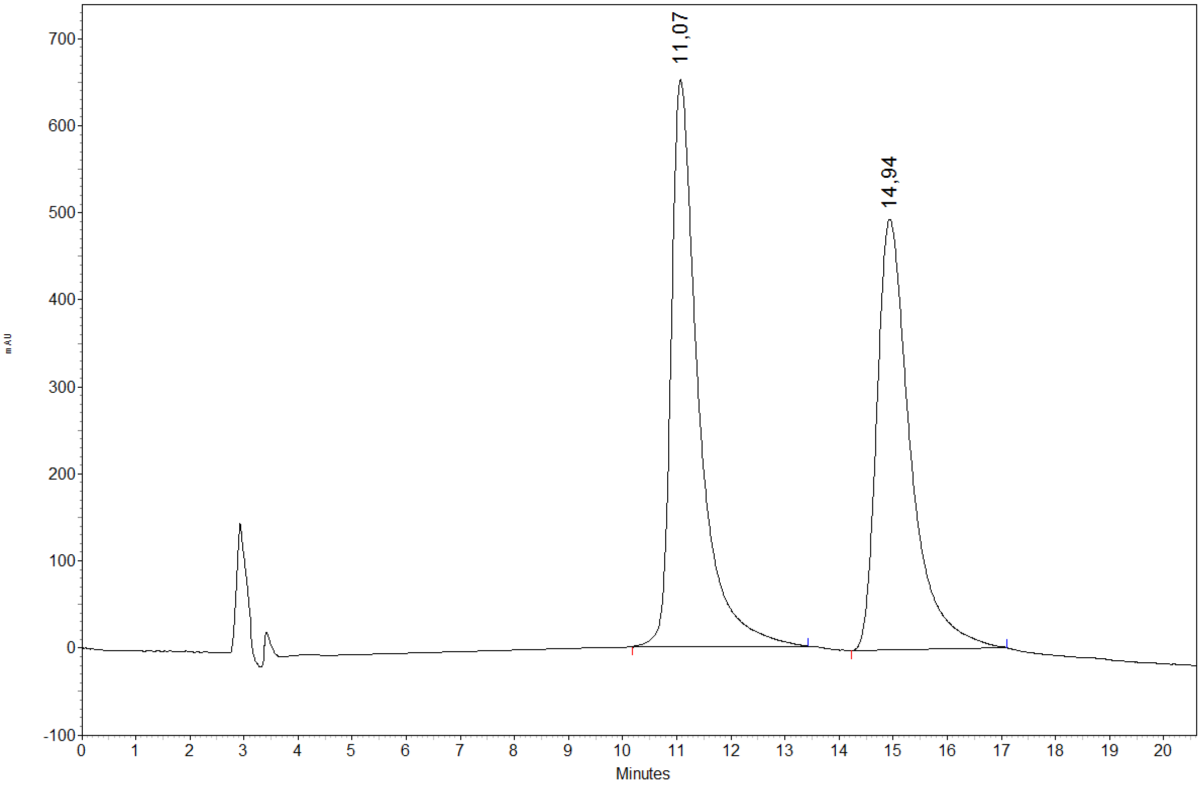

Figure 3. Chromatogram of racemic fenoterol on CHIRALLICA PST-4. Mobile phase was hexane/EtOH/DEA = 80/20/0.1, the flow rate was 1.0 ml/min, 220 nm. The retention time of the peak is given above the corresponding peak

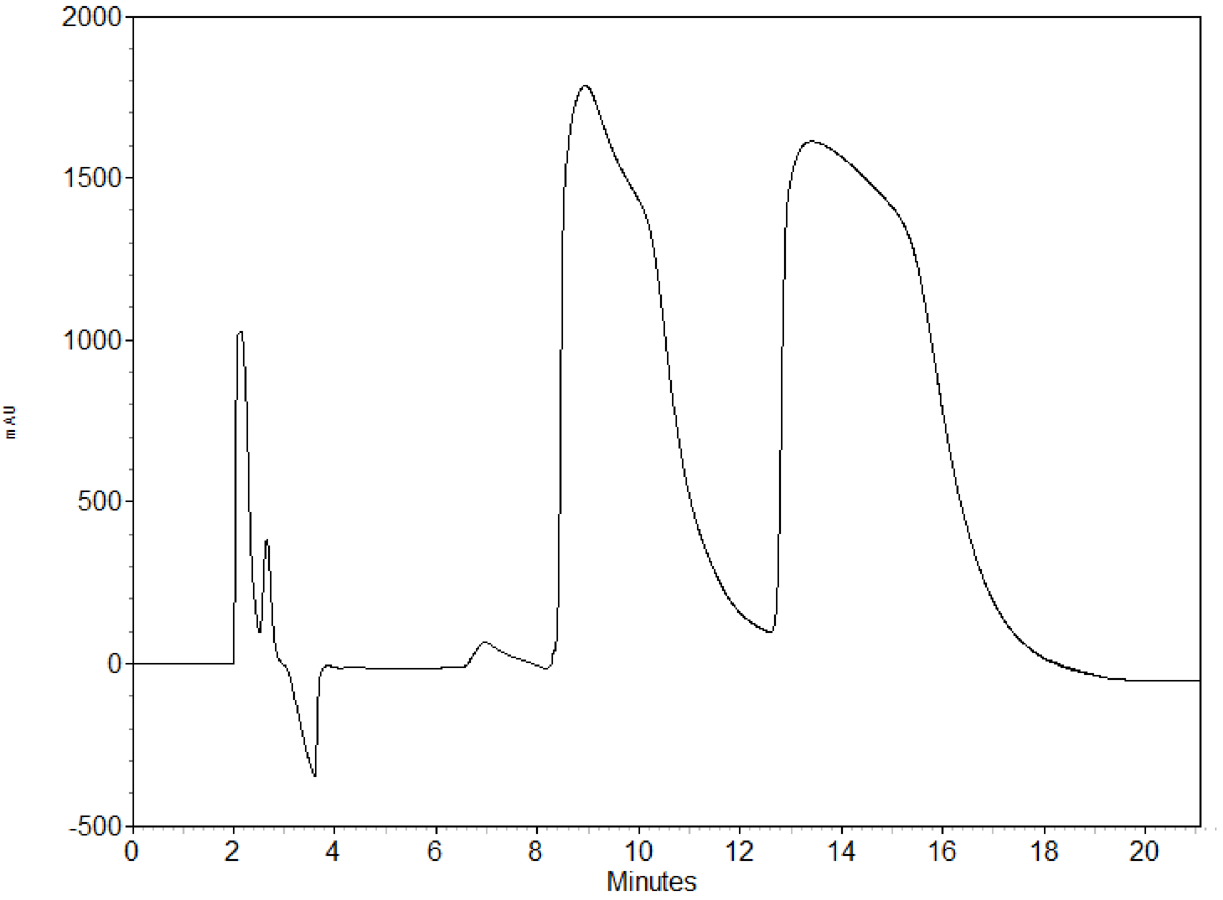

Figure 4. Chromatogram obtained for preparative separation of fenoterol enantiomers on CHIRALLICA PST-4 column. Column dimensions: 250 mm x 8 mm i. d., mobile phase hexane/EtOH/DEA = 85/15/0.1, the flow rate of 5 mL/min, 220 nm.

a)

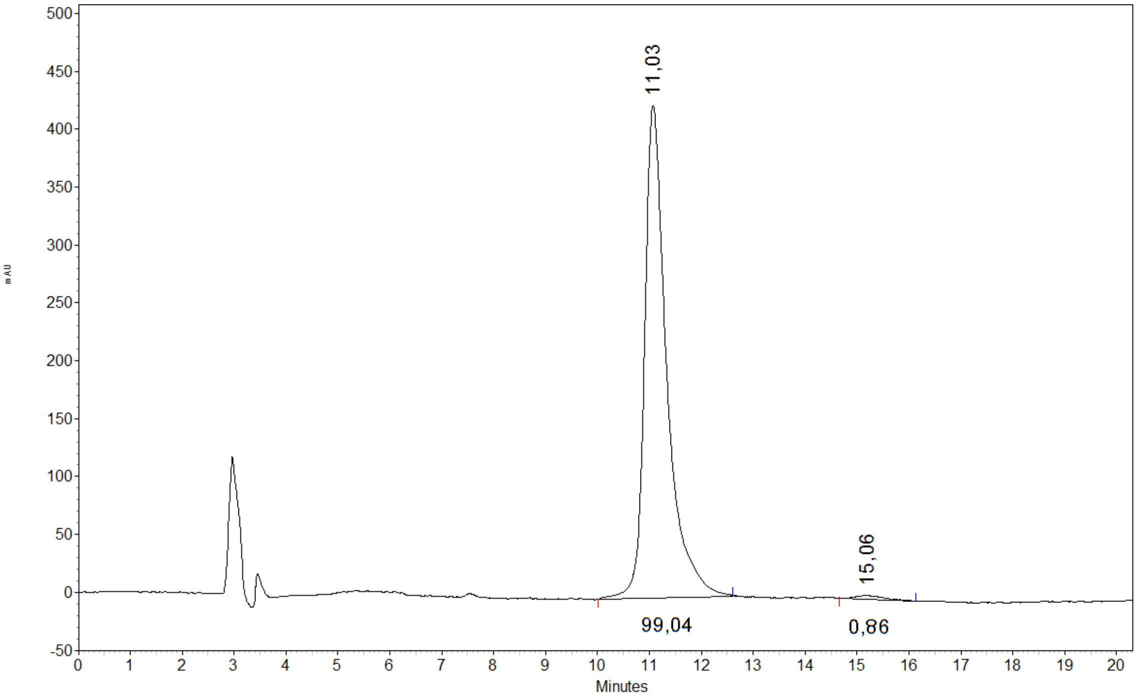

b)

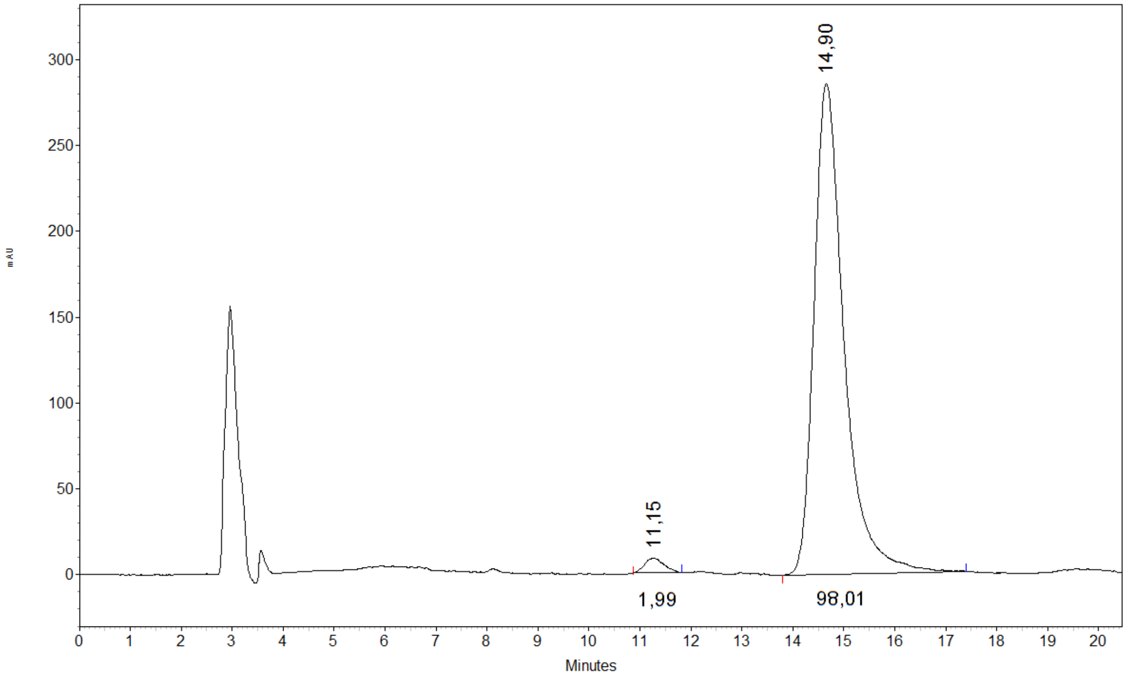

Figure 5. Determination of *ee* for a) (*S*, *S*)- and b) (*R*, *R*)-fenoterol on CHIRALLICA PST-4. Mobile phase was hexane/EtOH/DEA = 80/20/0.1, the flow rate was 1.0 ml/min, 220 nm. The retention time of the peak is given above the corresponding peak and area percentage is given below.

a)

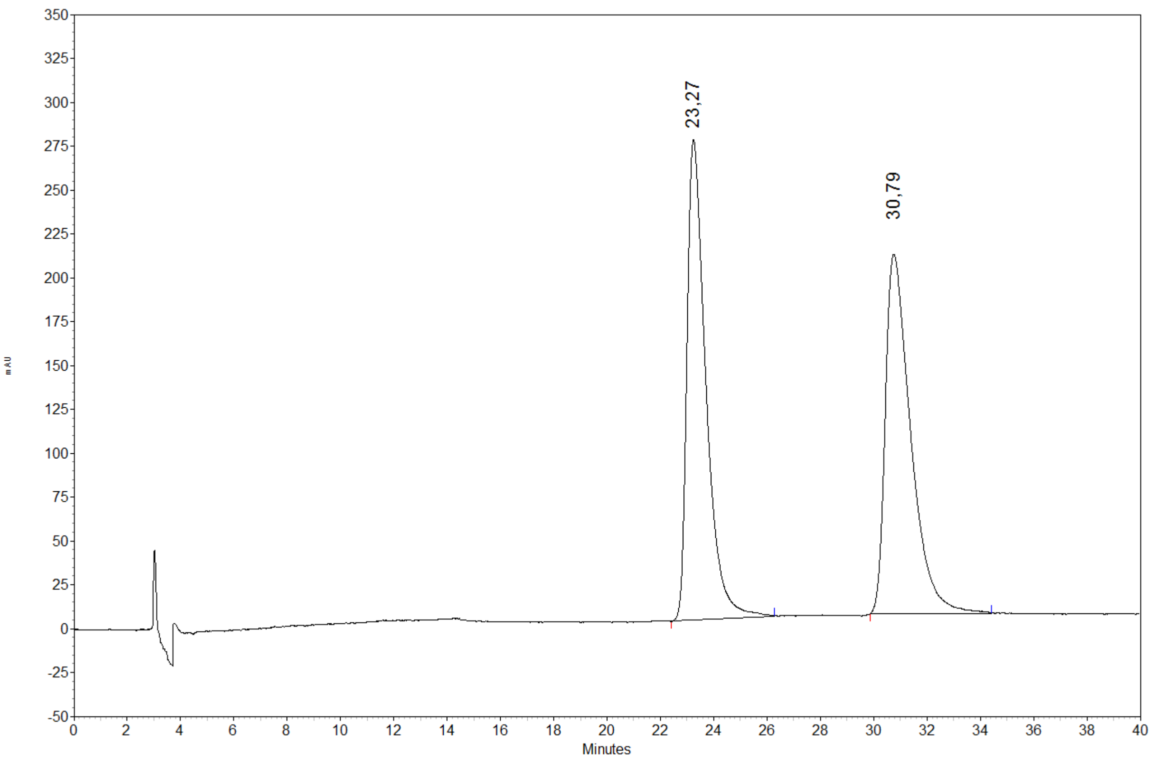

b)

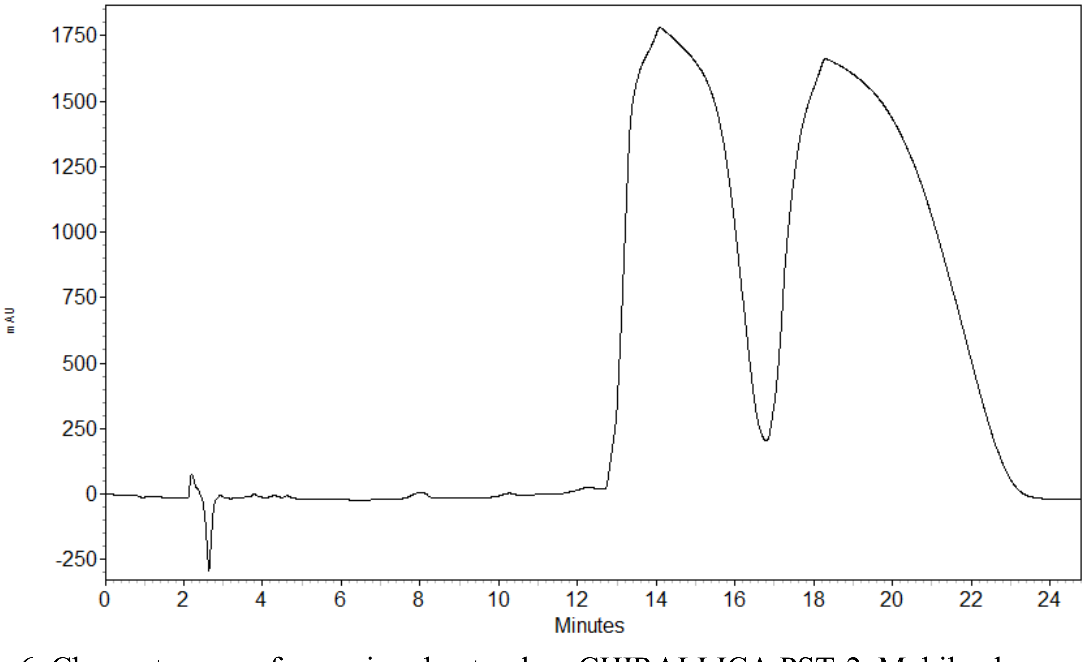

Figure 6. Chromatogram of racemic salmeterol on CHIRALLICA PST-2. Mobile phase was hexane/EtOH/DEA = 90/10/0.1, 220 nm, a) analytical column (dimensions: 250 mm x 4.6 mm i. d.) with the flow rate 1.0 ml/min, b) semi-preparative column (dimensions: 250 mm x 8 mm i. d.) with the flow rate 4.0 ml/min. The retention time of the peak is given above the corresponding peak.

a)

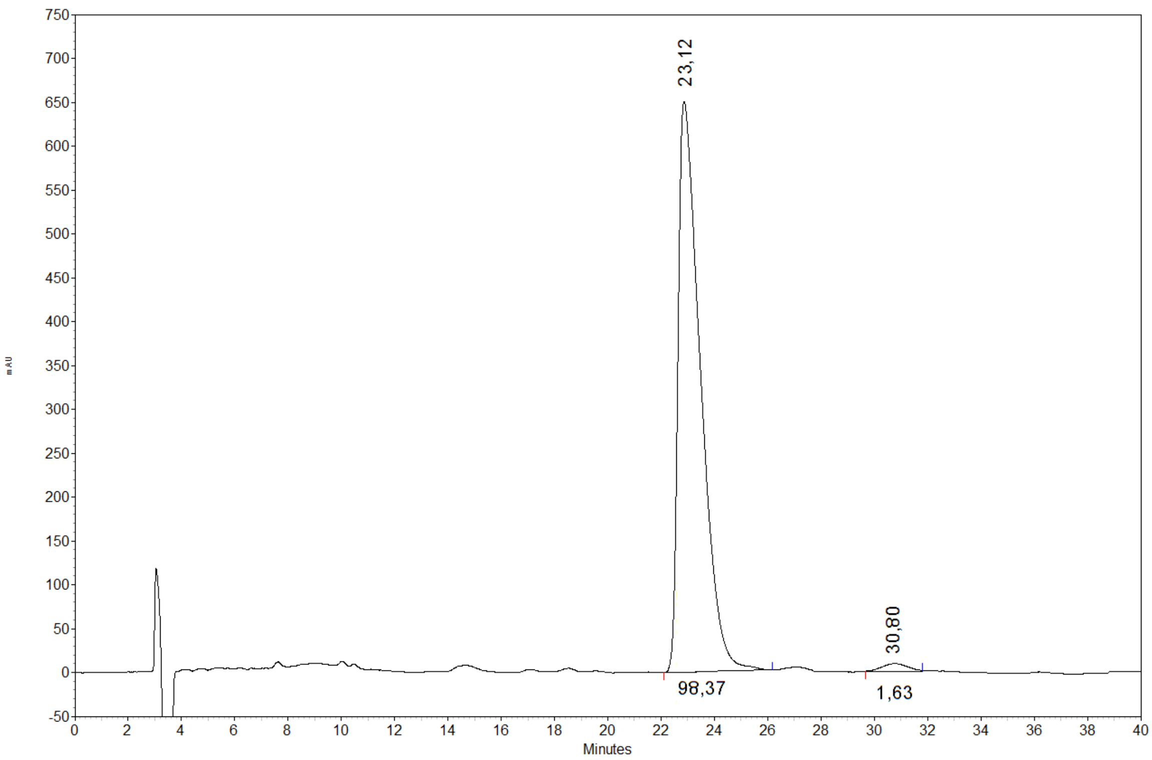

b)

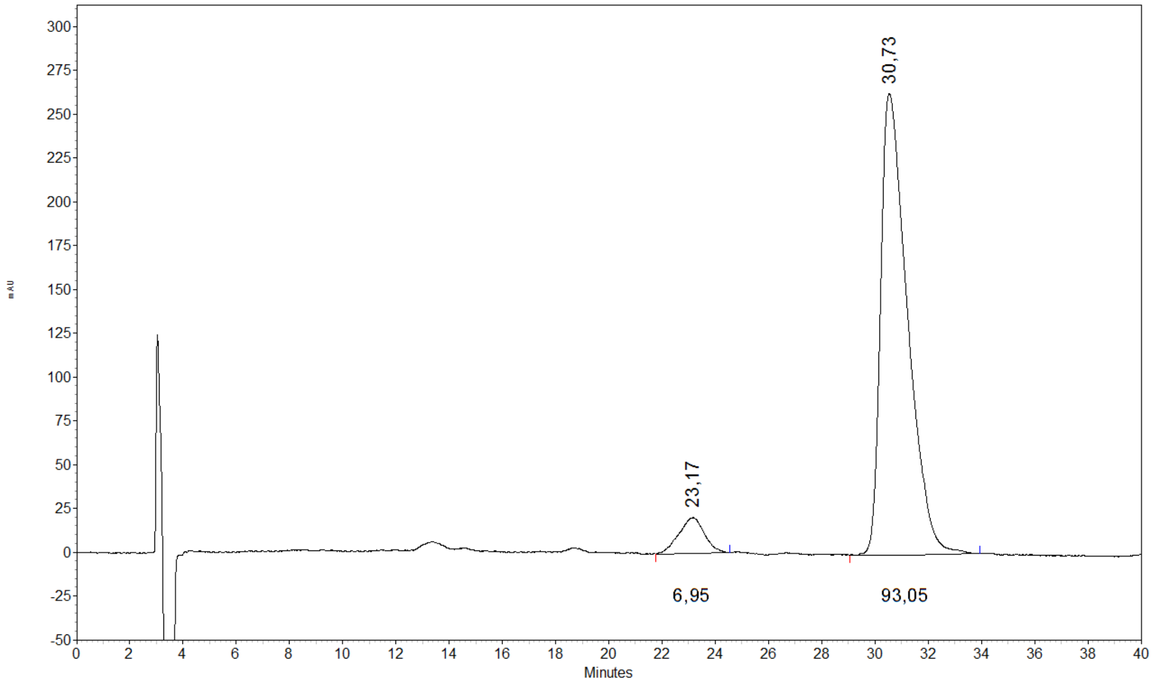

Figure 7. Determination of *ee* for a) (*R*)- and b) (*S*)-salmeterol on CHIRALLICA PST-2. Mobile phase was hexane/EtOH/DEA = 90/10/0.1, the flow rate was 1.0 ml/min, 220 nm. The retention time of the peak is given above the corresponding peak and area percentage is given below.
